# Supplementary material for: RNA-directed activation of cytoplasmic dynein-1 in reconstituted transport RNPs
Source: eLife. 2018 Jun 26;7:e36312. doi: 10.7554/eLife.36312 (PMC6056234; doi:10.7554/eLife.36312)
Supplement: Supplementary file 1. [file elife-36312-supp1.docx]

**Supplementary Table 1. Expected labelling combinations of SNAP::BICD2 or Egl::SNAP if there are two copies of the SNAP-tagged polypeptide per complex.**

| **Number of dyes per SNAP-tagged polypeptide^1^** | | | |
| --- | --- | --- | --- |
| 0.9 | | | |
|  | | | |
| **Probability polypeptide unlabelled** | | **Probability polypeptide labelled** | |
| 0.1 | | 0.9 | |
|  | | | |
| **Proportion of all complexes with two labelled polypeptides** | | | |
| TMR + A647 | TMR + TMR | | A647 + A647 |
| (0.9 x 0.9) x 0.5 = 0.405 | (0.9 x 0.9) x 0.25 = 0.2025 | | (0.9 x 0.9) x 0.25 = 0.2025 |
|  | | | |
| **Proportion of all complexes with one labelled polypeptide and one unlabelled polypeptide** | | | |
| TMR | | A647 | |
| ((0.9 x 0.1) + (0.1 x 0.9)) x 0.5 = 0.09 | | ((0.9 x 0.1) + (0.1 x 0.9)) x 0.5 = 0.09 | |
|  | |  | |
| **Proportion of all complexes with no labelled polypeptides** | | | |
| 0.1 x 0.1 = 0.01 | | | |
|  | | | |

^1^Determined by spectrophotometric analysis
