# Supplementary material for: RNA-directed activation of cytoplasmic dynein-1 in reconstituted transport RNPs
Source: eLife. 2018 Jun 26;7:e36312. doi: 10.7554/eLife.36312 (PMC6056234; doi:10.7554/eLife.36312)
Supplement: Supplementary file 2. [file elife-36312-supp2.docx]

**Supplementary Table 2. Observed outcome of dual labelling experiments with SNAP::BICD2 or Egl::SNAP vs expectation for two SNAP-tagged polypeptides per complex**

| **Signal** | **Observed^1^ (Mean ± SD) *SNAP::BICD2,***  ***Figure 7C*** | **Observed^1^ (Mean ± SD) *Egl::SNAP***  ***Figure 7D*** | **Expected^2^** |
| --- | --- | --- | --- |
| TMR+A647 | 0.3924 ± 0.049 | 0.3743 ± 0.074 | 0.405 |
| TMR | 0.3262 ± 0.066 | 0.3247 ± 0.072 | 0.2025 + 0.09 = 0.2925 |
| A647 | 0.2814 ± 0.062 | 0.3010 ± 0.061 | 0.2025 + 0.09 = 0.2925 |

^1^ Only motile puncta were analysed

^2^ See Supplementary Table 1
