# Supplementary material for: RNA-directed activation of cytoplasmic dynein-1 in reconstituted transport RNPs
Source: eLife. 2018 Jun 26;7:e36312. doi: 10.7554/eLife.36312 (PMC6056234; doi:10.7554/eLife.36312)
Supplement: Supplementary file 3. [file elife-36312-supp3.docx]

**Supplementary Table 3. Correction of Cy3-*hairy* + Cy5-*hairy* RNA mixing results (Figure 7G) for the proportion of RNA molecules containing no dye.**

| **Number of dyes per RNA molecule^1^** | | | |
| --- | --- | --- | --- |
| Cy3-*hairy*: 2.6  Cy5-*hairy*: 4.0 | | | |
|  | | | |
| **Number of dyes per nucleotide^2^** | | | |
| Cy3-*hairy*: 2.6 ÷ 730 = 0.00356  Cy5-*hairy*: 4.0 ÷ 730 = 0.00548 | | | |
|  | | | |
| **Probability RNA molecule unlabelled** | | **Probability RNA molecule labelled** | |
| Cy3-*hairy*: (1 – 0.00356)^730^ = 0.074 | | 1 – 0.074 = 0.926 | |
| Cy5-*hairy*: (1 – 0.00548)^730^ = 0.018 | | 1 – 0.018 = 0.982 | |
|  | |  | |
| **Proportion of motile complexes containing two RNAs that are both labelled^3^** | | | |
| Cy3 + Cy5 | Cy3 + Cy3 | | Cy5 + Cy5 |
| (0.926 x 0.982) x 0.50 = 0.455 | (0.926 x 0.926) x 0.25 = 0.214 | | (0.982 x 0.982) x 0.25 = 0.241 |
|  | | | |
| **Proportion of motile complexes containing two RNAs that have one or both RNAs unlabelled^3^** | | | |
| 1 – (0.455 + 0.214 + 0.241) = 0.09 | | | |
|  | |  | |
| **Observed proportion of motile RNA puncta containing both Cy3 and Cy5^4^** | | | |
| 0.1365 | | | |
|  | | | |
| **Proportion of motile RNA puncta containing two RNA molecules that are both labelled^5^** | | | |
| 0.1365 x 2 = 0.273 | | | |
|  | | | |
| **Proportion of motile RNA puncta that contain two RNA molecules (after correction for proportion of complexes that have one or both RNAs unlabelled)** | | | |
| 0.273 ÷ (1 – 0.09) = 0.30 | | | |

**^1^** Determined by spectrophotometric analysis

^2^ *hairy* RNA is 730-nt long

^3^ For simplicity, the expected proportions of complexes containing two RNAs in which there is one Cy3-labelled RNA or one Cy5-labelled RNA, or two unlabelled RNAs, are not shown separately.

^4^ From Figure 7G

^5^ Cy3 + Cy5, Cy3 + Cy3 or Cy5 + Cy5
